# Incomplete Patient Medication Histories Pose a Significant Barrier to Finding the Best Treatment Choices in Outpatient Community Care

**DOI:** 10.7759/cureus.101543

**Published:** 2026-01-14

**Authors:** Igor Wilderman, Olga Pugacheva-Zingerman, Bethany Jacobson

**Affiliations:** 1 Pain Physician, Wilderman Medical Clinic, Thornhill, CAN; 2 Biological Sciences, Canadian Centre for Clinical Trials, Thornhill, CAN; 3 Kinesiology, Wilderman Medical Clinic, Thornhill, CAN

**Keywords:** database, decision making, drugs, electronic medical records, failed drugs, medication history, medication therapy management, pharmacogenetic testing, referral and consultation, treatment

## Abstract

When physicians consult patients with chronic conditions, they often need to make treatment decisions. Pharmacological strategies are based on different sources, including complete medication histories. However, a physician’s access to a patient’s medication history is usually limited to current medications and known allergies. This editorial examines gaps in outpatient medication documentation and argues for systematic improvements in how medication response data are collected and shared. We propose adding medication-related information to a patient’s medical records that would facilitate determining effective treatment strategies, as these data are often neither collected nor presented in the medical history. The minimum required information includes data about medications discontinued due to adverse reactions, ineffectiveness, nonadherence, or excessive costs. In selected clinical contexts, pharmacogenetic testing may assist with treatment decision-making by identifying patients at higher risk of non-response or adverse effects. Based on a patient’s genetic variations in drug metabolism, pharmacogenetic results may allow physicians to anticipate treatment response and personalize guideline-based therapy for chronic conditions. In this article, we discuss the absence of this necessary information in medical records and its implications for selecting safe and effective treatments across various medical specialties. We propose implementing structured documentation of medication failures and, where appropriate, integrating pharmacogenetic data within privacy-secured databases to support individualized outpatient care. Having more complete pharmacological data for each patient, including drugs previously tried without success or discontinued for various reasons, and ideally a genetic metabolic profile, could help clinicians identify the most appropriate drugs on an individual basis.

## Editorial

Incomplete medication histories are a widespread and well-documented challenge in patient care, including in Ontario, Canada. A retrospective cohort study published in 2015 assessing the accuracy of patient medications recorded in electronic health records found one or more medication discrepancies in 76.9% of patients for active medications alone, citing care transitions as a particularly vulnerable time for these errors to occur [1]. Alqenae FA et al. performed a systematic review of available data on medication errors, noting that incomplete or inaccurate medication histories are associated with an increased risk of adverse drug events and suboptimal treatment outcomes. They found that “[f]or adult patients, the median rate of medication errors and unintentional medication discrepancies following discharge was 53% and 50% respectively” [2]. Although these data primarily reflect transitions from hospital to community care, they highlight vulnerabilities in medication documentation that persist into outpatient follow-up and chronic disease management. In patients with chronic disease and multimorbidity, these discrepancies are amplified by polypharmacy, fragmented care, and limited compatibility between EMR systems [2]. Despite the recognized importance of longitudinal medication data, most outpatient records remain focused on active prescriptions and allergies, and omit structured documentation of prior medication failures, intolerances, nonadherence, or cost-related discontinuation.

Physicians refer to a patient’s medical history and assessment results when developing pharmacological strategies for chronic and/or multimorbid patients. Treatment decisions are also based on current disease-specific guidelines and algorithms, the presence of comorbidities, and concomitant medications. Thus, finding the most effective pharmacological treatment is a complex and lengthy process requiring trial and error, which is frustrating and exhausting for both patient and physician. This process often involves collaboration between several clinical professionals and requires accurate recording and sharing of information. However, specific medication-related information is frequently not collected, recorded, or shared between clinical professionals, even though this knowledge could facilitate the most effective treatment decisions for individual patients. This information can include complete medication histories, details of medication discontinuation or failure, and results of pharmacogenetic testing.

Medication histories are not collected and not recorded

In clinical practice, incomplete medication histories frequently limit access to treatment-relevant information. One of the authors (IW), a practicing pain management physician, routinely encounters referrals with incomplete medication documentation, creating barriers to identifying effective treatment strategies and improving patient outcomes. Most referrals lack comprehensive historical medication details, and patients are often unable to accurately recall prior therapies or reasons for discontinuation. In addition, prior medication details are most often neither retained nor reported by the referring physician. Even medical records with meticulous medication histories often lack critical information to determine the most appropriate subsequent treatment course. For example, it is common practice to record details of medication discontinuation due to an allergic reaction; however, this is only one of several possible reasons to interrupt a treatment. Adverse reactions, unwanted side effects, or ineffectiveness, possibly due to nonadherence to prescribed regimens or suboptimal doses, are other frequent reasons for patients to discontinue their medications. It has been estimated that approximately 50% of patients do not adhere to their prescribed medication regimens; in addition, multimorbidity and polypharmacy further reinforce nonadherence behaviors [3]. Knowing why certain medications were discontinued has a substantial impact on clinical choices for an individual patient. It could indicate the risk of side effects and the patient’s financial burden, and it could also affect the speed of treatment optimization and ultimately the patient’s mental health and quality of life.

Details about medication failures are rarely recorded in the cumulative patient profile (CPP) of the medical chart. In fact, the standard CPPs of most electronic medical record (EMR) software lack an option to record medication failure details. In clinical practice, one of the authors (IW) implemented an additional data field to record medication failure information. This implementation is a local software modification; therefore, the information is not transferable to other healthcare providers. Moreover, the flexibility to customize EMR software may not be offered by all EMR systems. Collecting this historical information is time-consuming and difficult, and should not be left to the discretion of individual physicians. Complete and detailed recording of a patient’s medication history should be standard practice, and it should be reflected in the clinical software accordingly. In addition, enhanced collaboration with pharmacists would be invaluable in capturing and transferring the details of medication histories.

Beyond informing individual clinical decisions, systematic documentation of medication non-response has the potential to generate clinically meaningful information. Patterns of non-response across drug classes, particularly in conditions such as chronic pain or migraine, where treatment efficacy varies widely, may reflect underlying genetic, metabolic, or disease-specific mechanisms rather than treatment failure alone. Consistent, detailed recording of non-responders could reveal important patterns, such as genetic influences on drug response or variations in how the disease presents.

Pharmacogenetic reports are rarely available

Pharmacogenetic testing also shows promise in supporting clinical treatment decisions. Recent advances in this field individualize the approach to choosing the most beneficial medications and dose regimens. Current drug-dosing strategies are developed based on population data; however, optimal doses vary widely within the population, due in part to genetic variations in drug metabolism. Pharmacogenetic testing can distinguish potential responders and non-responders and identify patients at higher risk of side effects or adverse drug reactions. These unexpected responses might occur if “genetically unsuitable” medications are prescribed based on standard drug-of-choice algorithms. However, pharmacogenetic testing is currently rarely used in clinical practice and is costly for patients; moreover, even if a patient has undergone this testing, the results may be dismissed by some members of their healthcare team. Overcoming the barriers to widespread implementation of pharmacogenetic testing is a critical step to improve patient care.

For chronic disease management, the impact of the availability and accessibility of patient-specific genetic information on drug response and metabolism could be pivotal and should not be underestimated [4]. In our practice, the use of pharmacogenetic testing considerably shortens the process of trial and error and helps to avoid predictable adverse events. A recently published study demonstrated that pharmacogenetic-guided care of patients with moderate or severe major depressive disorder was associated with 37% fewer patients with refractory depression over 20 years [5]. From a health economics perspective, the same study calculated that implementing pharmacogenetic testing for adult patients with major depressive disorder could save the health system in British Columbia (Canada) $956 million [5].

Taken together, as research in this field continues and more genetic variants impacting drug response are discovered, this knowledge will have an increasingly important role in determining pharmacological strategies and personalizing care for patients with chronic diseases, particularly in conditions where treatment response varies significantly. For example, in several therapeutic areas, including pain management and migraine prevention, clinical trials demonstrate substantial proportions of non-responders, even to guideline-recommended first-line therapies. These response distributions suggest that population-based treatment algorithms may obscure clinically relevant biological differences among patients. Integrating structured non-response data with pharmacogenetic testing could help distinguish genetic contributors to treatment resistance from other factors such as disease variation or medication adherence.

Medication histories are not shared among clinicians

Currently, in routine outpatient practice, accurate and up-to-date details regarding a patient’s response to a medication are typically not accessible through referrals or electronic databases. As a result, patients with multiple chronic conditions, whose clinical management is already complex and fragmented across different physicians, may suffer for years while waiting for the optimal treatment to be identified. Clinicians need instant access to complete patient medication records to address this problem and potentially determine the most beneficial treatment options for an individual patient. In Ontario, Canada, instant access is currently available for laboratory reports via the Ontario Laboratories Information System (OLIS); moreover, selected patients’ records (limited to hospital visits and hospital diagnostic reports, whereas records from outpatient community clinics and private diagnostic facilities are not included) and some information about publicly funded dispensed medications can be accessed via the ConnectingOntario Clinical Viewer. These real-time connected systems are critical for enabling instant access to patient data that can inform clinical decisions. However, these systems are limited to information provided by hospitals and government-funded dispensaries and lack detailed medication histories, including information such as reasons for medication failures, unfilled prescriptions, low medication adherence, ineffectiveness, and side effects or adverse reactions. Taken together, these gaps in documentation, data sharing, and pharmacogenetic integration represent a modifiable barrier to more effective outpatient care. While implementing such changes would require investment, workflow adaptation, and strong governance frameworks, similar centralized health data systems demonstrate that such integration is achievable.

Moving forward

Complete drug response information is fundamental to facilitating personalized, integrated care for patients with chronic diseases, and access to it should be incorporated into routine clinical practice. The creation of a centralized database, similar to existing systems like OLIS for laboratory data, containing detailed medication records from physicians and pharmacists, patient medication outcomes, and results of pharmacogenetic testing, would enable access to complete medication histories. Such systems would require robust privacy protections and patient consent mechanisms to ensure appropriate data sharing while maintaining confidentiality. Furthermore, we suggest physician education and training to improve uptake and continued use of online systems. Together, these changes would support safer, more efficient, and more individualized prescribing practices in outpatient care. In addition to improving individual patient care, standardized collection of medication response and non-response data would create opportunities for large-scale observational analyses, quality improvement initiatives, and future prospective studies. Such data could inform guideline refinement, identify patient subgroups at higher risk of treatment failure, and support more efficient and ethical prescribing practices by reducing unnecessary trial-and-error exposure.
